# Makeen Document Control System: Development, Standards-Based Validation, and Early Multisite Implementation of a Saudi-Built Laboratory Document Control Platform

**DOI:** 10.7759/cureus.111945

**Published:** 2026-07-02

**Authors:** Kaneez Zamir, Abdulaziz Johani, Omar Qassas, Abdulrahman Aboud, Bilal Waheed

**Affiliations:** 1 Quality and Accreditation Department, Saud Abdulaziz Al Shalan Company, Riyadh, SAU; 2 Information Technology Department, Saud Abdulaziz Al Shalan Company, Riyadh, SAU

**Keywords:** aabb, cap, cloud computing, digital transformation, document control system, iso 15189, iso 17025, laboratory accreditation, quality of life program, saudi arabia 2030 vision

## Abstract

Background

Document control is a core requirement of laboratory quality management and accreditation, including under the College of American Pathologists (CAP), International Organization for Standardization (ISO) 17025, ISO 15189, the Association for the Advancement of Blood & Biotherapies (AABB), and the Central Board for Accreditation of Healthcare Institutions (CBAHI). Before 2024, many Saudi laboratories relied on paper-based systems or imported platforms that lacked Arabic-language functionality, local hosting, and explicit alignment with Saudi regulatory and governance requirements.

Objective

To describe the development, standards-based internal validation, and early multisite implementation of the Makeen Document Control System (Makeen DCS), a Saudi-built laboratory document control platform designed for accreditation-oriented use in Saudi Arabia, and to assess early feasibility and user-perceived acceptability rather than comparative effectiveness.

Methods

This descriptive implementation and feasibility study was conducted from October 2024 to January 2026. System requirements were derived from CAP, ISO 17025, ISO 15189:2022, AABB, CBAHI, FDA 21 CFR Part 11, and Saudi Personal Data Protection Law (PDPL) requirements and translated into software specifications. Development followed staged requirements definition, prototyping, workflow refinement, internal validation, and pilot deployment. Internal validation used 126 structured test cases across user access, document creation and upload, approval workflow, version control, retrieval, audit trail behavior, and administrative functions. Early implementation was undertaken in seven purposively selected pioneer laboratory sites. User feedback was collected through an internally developed implementation survey completed by eight participants from pilot sites.

Results

The Makeen DCS provided end-to-end document lifecycle management, role-based access control, bilingual Arabic-English interfaces, dynamic master document lists, searchable retrieval, audit-oriented reporting, and a personnel-documentation module. The system supported both Saudi-hosted cloud deployment and on-premises implementation. Functional alignment was mapped against selected CAP, ISO 17025, ISO 15189:2022, CBAHI, AABB, FDA Part 11, and PDPL requirements. Among 126 validation test cases, 109/126 (86.5%) passed on first execution, and 17/126 (13.5%) failed initially; all failed items were corrected and retested, resulting in a final pass rate of 100% (126/126). Early deployment was achieved across seven diverse sites, of which six were fully active, and one remained partially implemented by study cut-off. Median active users across sites were 80 (range: 30-200), median configured departments were 10 (range: 5-15), and median migrated or newly created controlled documents were 76 (range: 10-274) across six sites with available data. User feedback was favorable, with a mean satisfaction score of 9/10, 7/8 (87.5%) respondents willing to recommend the platform, and 8/8 (100.0%) perceiving improved inspection readiness and reduced manual or paper-based workload.

Conclusions

Makeen DCS was feasible as a Saudi-built/bilingual/locally hosted lab platform for accreditation workflows. Internal validation, pilot use, and small-sample feedback show readiness/acceptability, not operational effectiveness; independent longitudinal evaluation is needed.

## Introduction

Document control is a foundational component of laboratory quality management because it determines whether policies, procedures, records, and related evidence are current, approved, retrievable, traceable, and consistently applied in practice. In accredited laboratory environments, document control is not merely administrative; it is part of the infrastructure through which laboratories standardize work, demonstrate compliance, maintain traceability, and support patient safety. This central role is reflected across major accreditation and regulatory frameworks, including the College of American Pathologists (CAP), International Organization for Standardization (ISO) 17025, ISO 15189:2022, and the Association for the Advancement of Blood & Biotherapies (AABB), all of which require controlled management of documented information and records as part of broader quality and competence systems [1-3].

Digitalization has increased the importance of document control as an operational rather than purely clerical function. In laboratory settings, electronic systems can support controlled approval workflows, version management, searchable retrieval, audit trails, and improved visibility of review status and accountability [4-6]. However, the value of digital document control depends on more than the conversion of paper to electronic format. In regulated environments, electronic systems must also align with requirements related to access control, record integrity, governance, and reliability, including principles reflected in FDA 21 CFR Part 11 and related quality-system expectations [7]. Accordingly, a document control platform for laboratories must be assessed not only in terms of usability, but also in relation to standards alignment, controlled workflow support, and implementation feasibility.

These issues are particularly relevant in Saudi Arabia, where accreditation expansion, healthcare reform, and digital transformation are advancing in parallel. Saudi evidence has shown that laboratory quality challenges are often linked not only to analytical issues but also to inconsistency in documentation, variable standardization, limited information-technology support, and gaps in implementation infrastructure [8-11]. Saudi institutions also operate within a distinctive governance context that includes national accreditation expectations, Arabic-language workflow needs, and increasing emphasis on domestic data handling under the Personal Data Protection Law (PDPL) [12,13]. For Saudi laboratories, therefore, a suitable document control platform must do more than provide generic document management functions; it must be usable within local regulatory, linguistic, and operational conditions.

Despite this need, there is limited published evidence on laboratory document control platforms developed and evaluated specifically for Saudi laboratory settings. Existing literature addresses accreditation readiness, organizational barriers, practitioner perceptions, and broader laboratory digital transformation, but not the design and early evaluation of a Saudi-built, bilingual, standards-mapped platform for laboratory document control [4-6,8-13]. This creates a practical and scholarly gap. The unanswered question is not simply whether digital document control is useful in principle, but whether a locally developed platform can be designed and internally validated in a way that aligns with the CAP, ISO 17025, ISO 15189, AABB, the Central Board for Accreditation of Healthcare Institutions (CBAHI), electronic-record expectations, and national data-governance priorities while remaining feasible for implementation in real Saudi laboratory settings.

To address this gap, Saud Abdulaziz Al Shalan Company (SASC) initiated the Makeen Document Control System (Makeen DCS) project in October 2024 as a Saudi-built laboratory document control platform intended for accreditation-oriented use. The primary aim of the present study is to describe the design, development, standards-based internal validation, and early multisite implementation of Makeen DCS in Saudi laboratory settings. The secondary aims are to examine its alignment with selected accreditation and regulatory requirements, assess the feasibility of early implementation across diverse laboratory sites, and describe preliminary user-perceived benefits and barriers. Importantly, the study was not designed as an independent comparative effectiveness evaluation and does not attempt to establish causal effects on workflow performance, accreditation outcomes, or patient-level endpoints.

Accordingly, this study addresses four focused questions: (1) Can Makeen DCS be mapped and internally validated against selected accreditation and regulatory requirements? (2) Is early implementation feasible across diverse Saudi laboratory settings? (3) What early user-perceived benefits and barriers emerge during implementation? (4) Which descriptive implementation outcomes can be demonstrated at this stage?

By answering these questions, the study contributes early implementation evidence on a Saudi-built, accreditation-oriented digital document control platform in a context where compliance, localization, and digital governance must be addressed together. This focus is consistent with current laboratory quality literature, which treats documents, records, information management, assessments, and continual improvement as core elements of quality-system architecture rather than peripheral administrative tasks [14-17].

## Materials and methods

Study design

This study was a descriptive implementation and feasibility study of the design, standards alignment, internal functional validation, and early pilot deployment of the Makeen DCS in selected Saudi laboratory settings. The project combined requirements engineering, iterative software development, standards-based internal validation, and early real-world implementation. The study period extended from October 2024, when formal requirements definition and system planning began, through January 2026, when validation and first-wave deployment had been completed. The study was intended to describe system development, internal functional readiness, and early implementation feasibility; it was not designed as an independent comparative effectiveness study. No formal implementation-science framework or effectiveness-evaluation framework was applied prospectively; therefore, the findings are reported as descriptive implementation and feasibility outcomes rather than as evidence of comparative effectiveness. No formal implementation-science framework or effectiveness-evaluation framework was applied prospectively; therefore, the findings are reported as descriptive implementation and feasibility outcomes rather than as evidence of comparative effectiveness.

Setting and stakeholders

The project was undertaken by SASC, a Saudi organization providing laboratory quality consultancy and technology solutions. The development context was informed by prior accreditation and consultation experience across Saudi laboratories, including work related to CAP, ISO 17025, ISO 15189, AABB, and CBAHI implementation. Stakeholders included the project lead, who oversaw requirements definition, compliance architecture, and validation planning; software developers and infrastructure engineers responsible for platform development and hosting; UI/UX designers responsible for the bilingual Arabic-English interface; and laboratory directors and quality managers from participating pilot sites who contributed to demonstrations, onboarding, and operational feedback. Requirements definition, feature refinement, and readiness review were conducted through structured internal meetings during the development and pre-deployment phases. Functional and validation testing were also conducted internally. No independent external validation body participated in this phase; accordingly, the work is presented as an internal implementation and feasibility evaluation.

Requirements analysis

System requirements were derived from a structured review of document-control-related expectations within major accreditation and regulatory frameworks relevant to Saudi laboratories. These included CAP GEN.20375 and COM.10000-10500, ISO 17025 and ISO 15189:2022 requirements related to control of documents, records, and information systems, CBAHI CLBB standards 08100 and 08200, AABB Quality System Essentials 6.0-6.1.9, FDA 21 CFR Part 11 requirements relating to electronic records and electronic signatures, and Saudi Personal Data Protection Law (PDPL) provisions relevant to data processing and cross-border transfer. Rather than relying on a single standard, requirements were synthesized thematically across these frameworks and translated into system functions intended to support controlled document approval, version management, archival retention, audit trail visibility, searchable retrieval, role-based access control, bilingual usability, data-hosting constraints, and personnel-documentation workflows. A document-control workflow model was created to visualize the desired lifecycle, approval structure, and permission logic. An informal review of existing document-control platforms was undertaken during requirements scoping to identify practical gaps relevant to Saudi laboratories, particularly Arabic-language usability, local hosting expectations, and alignment with national accreditation workflows. Because this review was not conducted using predefined independent comparison criteria, comparative product claims are not presented as study outcomes.

The design logic of Makeen DCS was also informed by recent literature on laboratory informatics and workflow digitization. Published reports have highlighted the role of informatics systems in supporting traceability, governance, and public safety [18], the importance of structured quality management in digital pathology operations [19], the feasibility of digitizing complex laboratory workflows [20], and the value of digital transformation in strengthening routine quality-assurance activities [21]. Although the present study was not designed as a comparative effectiveness study, these reports provided relevant implementation context for the platform’s architecture, validation focus, and workflow-oriented development pathway.

Development process

Development followed an iterative staged pathway consisting of requirements definition, prototype development, workflow refinement, validation/readiness review, and early pilot deployment. During the initial phase (October-December 2024), the software requirements specification was reviewed and refined through structured project meetings involving the quality and development teams. During the prototype phase (January-May 2025), a first working version of the platform was built with core functions including user management, document creation and upload, lifecycle control, role-based access, search, and reporting views. During the refinement phase (June-July 2025), the system was expanded through iterative internal review, including further development of bilingual presentation, searchable repositories, audit-trail functionality, role-based administration, reporting features, and the personnel-documentation area ("My Workspace"). Infrastructure deployment in Dammam and preparation for data migration also began during this stage. During the extended testing and pre-deployment phase (August-November 2025), broader functional testing was performed, identified defects were logged and corrected, and workflows were re-reviewed before rollout. Formal validation and approval were completed between December 2025 and January 2026, after which the platform was approved for early laboratory use. Meeting minutes and action lists were retained as part of the internal development trail.

Validation protocol

Internal validation was conducted using predefined structured test cases mapped to major compliance-related functions of the system. The validation log for each test case recorded the preconditions and required configuration, the user role and action to be performed within Makeen DCS, the expected system response or output, the observed result, and a pass/fail judgment. Validation covered the following functional domains: user access and permissions, document creation and upload, review and approval workflow, version control, retrieval and visibility, status changes and archival behavior, audit-trail behavior, role-based administration, and implementation-support functions. A test case was classified as passing only when observed system behavior matched the predefined expected result without deviation. When a deviation was identified, corrective action was taken, and the affected test case was re-executed against the same predefined expectation until the expected result was achieved. In total, 126 validation test cases were executed across these domains. Validation was conducted internally by the project team to establish functional readiness of the release used for pilot deployment; no independent external validation body participated.

Early implementation and data collection

Following internal validation, the Makeen DCS was deployed on cloud infrastructure located in Dammam, Saudi Arabia. Site information forms were collected from candidate laboratories to support the configuration of organizational hierarchies, departments, user roles, and document categories. A first-wave pilot implementation was then undertaken in seven purposively selected pioneer sites chosen as early adopters on the basis of implementation readiness, willingness to participate, availability of local focal persons or site administrators, and the aim of representing different laboratory contexts. These sites included laboratories in Jazan, Makkah, Aseer/Abha, Riyadh, and an internal implementation environment.

Implementation data were descriptive and derived from routine deployment records rather than from a controlled comparison design. The principal implementation outcomes reported in this study were site activation status, number of active users, number of configured departments, and number of migrated or newly created controlled documents. Objective operational performance measures such as document retrieval time, approval turnaround time, overdue review rates, and document-related nonconformities were not uniformly collected during this early phase and therefore were not available as standardized quantitative outcomes. Thus, the implementation outcomes should be interpreted as predefined descriptive deployment indicators for this report, not as operational effectiveness endpoints.

Early user feedback was collected through an internally developed implementation-feedback survey completed by eight purposively selected respondents from pilot sites, including laboratory directors, quality managers, document-control personnel, and staff users. The survey captured overall satisfaction, willingness to recommend the platform, perceived inspection readiness, perceived reduction in manual or paper-based workload, training and support experience, willingness to continue use under a paid adoption model, accreditation or audit frameworks supported by the platform, and free-text comments. Because the survey was internally developed, not externally validated, and completed by a small purposive sample, findings were analyzed descriptively and were intended to characterize early implementation experience rather than support inferential analysis or generalizable effectiveness claims.

Ethical and data protection considerations

The project focused on system design, standards alignment, internal validation, and organizational implementation rather than on patient-level research. No patients were enrolled, no clinical interventions were performed, and no identifiable patient data were analyzed. The staff feedback component is therefore best characterized as service-evaluation or quality-improvement feedback collected during early implementation rather than formal human-subject clinical research. Formal research ethics committee approval was not sought because the project involved system implementation, internal validation, and aggregate staff feedback without patient enrolment, clinical intervention, or identifiable patient-level data. Feedback findings are presented in aggregated form without direct personal identifiers. Data protection and governance were addressed through Saudi-based hosting, role-based access control, and use of non-persistent data in demonstration environments, in line with the platform's stated PDPL-oriented design principles.​

## Results

System features and architecture

Makeen DCS was implemented as a web-based document control platform for laboratory governance in Saudi Arabia. The deployed architecture comprised application and database servers hosted in Dammam, Saudi Arabia, secure, browser-based access, and role-based authentication controls. Across development and early deployment, the platform provided controlled workflows for document lifecycle management, audit visibility, bilingual operation, and reporting functions.

The platform supported document creation or upload with defined metadata, followed by structured review and approval workflows. Approved documents were distributed to end users as controlled versions, while subsequent revisions generated new version records. Superseded documents were retired and archived rather than deleted, thereby preserving traceability and historical access. Each system action generated an audit record containing the user identity, timestamp, and action type, supporting traceability and record integrity.

Access was role-dependent. The system supported system administrators, site administrators, folder administrators, primary authors, inspectors, and standard staff users. These roles governed permissions related to document creation, editing, approval, distribution, and viewing. Additional controls could be applied to restrict copying, printing, or exporting of controlled documents according to local governance requirements.

A bilingual Arabic-English interface was implemented across administration pages, workflow screens, and reporting views. The system maintained a master document list showing document title, identifier, department, document type, version, approval status, responsible author, and review date. Version history remained accessible for archived documents, and the search function supported retrieval by title, keyword, department, type, and date range. The Makeen DCS also included reporting views for pending approvals, overdue reviews, user acknowledgements, and document history, as well as a personnel-documentation area ("My Workspace") for storage and oversight of licenses, training records, competency records, job descriptions, curricula vitae, and certificates.

In its default configuration, the system was hosted within Saudi Arabia, with no routine cross-border transfer of data. A cloud-hosted model was used for early deployment, while an on-premises option was also available for institutions requiring internal infrastructure control. This deployment model was consistent with the broader accreditation and digital-governance context in Saudi Arabia, where CAP-based accreditation remains an important reference model for laboratory quality assurance [22], ISO/IEC 17025 accreditation has been associated with measurable quality gains in Saudi laboratories [23], and calls for standards unification in forensic laboratory practice reinforce the need for structured and interoperable quality systems in regulated specialist environments [24]. At the national level, Saudi Arabia's digital transformation agenda under Vision 2030 has emphasized coordinated digital infrastructure [25], while recent work on data sovereignty and the Saudi Personal Data Protection Law has highlighted the importance of local governance and controlled data handling [26,27]. Related Saudi studies on accreditation automation and digitally secured record systems further support the relevance of standards-aware digital platforms [28,29], while ISO remains a central framework for standardization across regulated systems [30].

Standards alignment and validation findings

The Makeen DCS was developed against document-control-related requirements drawn from CAP, ISO 17025, ISO 15189:2022, CBAHI, AABB, FDA 21 CFR Part 11, and Saudi PDPL. Table 1 summarizes the alignment exercise between selected standards clauses and corresponding Makeen DCS functions. Across these domains, the platform was configured to support controlled approval workflows, version control, archival retention, traceable retrieval, role-based access, audit logging, and Saudi-hosted data governance.

**Table 1:** Examples of standards alignment Abbreviations: AABB, Association for the Advancement of Blood & Biotherapies; CAP, College of American Pathologists; CBAHI, Central Board for Accreditation of Healthcare Institutions; CLBB, Clinical Laboratory and Blood Bank; DCS, Document Control System; FDA, Food and Drug Administration; ISO, International Organization for Standardization; ISO/IEC, International Organization for Standardization/International Electrotechnical Commission; PDPL, Personal Data Protection Law.

Standard/clause	Requirement (summary)	Makeen DCS capability (summary)
CAP GEN.20375 [1,22]	Access to current documents; archival of obsolete versions	Version control, read‑only access, automatic archival of retired docs
CAP COM.10000-10500 [1,22]	Document creation, review, approval workflows	Structured workflows with role‑based sign‑off and audit logs
ISO 17025 [1,23,30]	Control of documents, records, and laboratory information	Controlled document approval, secure storage, traceable retrieval, version control, and role-based access
ISO 15189:2022 §8.3 [2,16]	Control of documented information	Approval workflow, master list, review reminders, distribution logs
ISO 15189:2022 §8.4 [2,16]	Control of records	Secure storage, traceable retrieval, retention and archival
ISO 15189:2022 §7.6.3 [2,16]	Information system management	Role‑based access, secure login, validation documentation
CBAHI CLBB 08100 [9,11]	Laboratory document control procedures	Full document lifecycle aligned with national requirements
CBAHI CLBB 08200 [9,11]	Record management and traceability	Comprehensive record keeping, timestamps, user identification
AABB QSE 6.0-6.1.9 [3]	Documents and records management	Controlled workflows and periodic review mechanisms
FDA 21 CFR Part 11 [7]	Electronic records and signatures	Audit trails, electronic approvals, data integrity controls
Saudi PDPL [12,27]	Data protection and data sovereignty	KSA‑based hosting, controlled access, no routine cross‑border transfer

Internal validation was performed using structured test cases covering major system functions. Validation domains included user access and permissions, document creation and upload, review and approval workflow, version control, retrieval and visibility, status changes, audit trail behavior, role-based administration, and implementation-support functions. Each test case was assessed against predefined expected outcomes, and failed items were corrected and re-tested until the expected result was achieved.

Across all validation domains, a total of 126 test cases were executed. Of these, 109/126 (86.5%) passed on first execution, and 17/126 (13.5%) failed initially. All initially failed items were corrected and re-tested, resulting in a final pass rate of 100% (126/126). These results support internal functional readiness of the validated release within the tested implementation domains, while remaining limited to internally conducted validation (Table 2).

**Table 2:** Validation execution summary Abbreviation: DCS, Document Control System

Validation metric	Value
Total validation test cases executed	126
Initially passed	109
Initially failed	17
Initial pass rate	86.5%
Retested after correction	17
Final passed	126
Final pass rate	100%

Early deployment and multisite implementation

Following validation, a first wave of seven pioneer sites was onboarded across Jazan, Makkah, Aseer, and Riyadh, together with the internal SASC laboratory environment. These sites were purposively selected as early adopters to represent varied Saudi laboratory contexts, including regional reference laboratories, forensic and toxicology services, poison control services, and an internal implementation site. Their contextual characteristics are summarized in Table 3.

**Table 3:** Contextual characteristics of pioneer implementation sites Abbreviations: CAP, College of American Pathologists; CBAHI, Central Board for Accreditation of Healthcare Institutions; ISO, International Organization for Standardization; ISO/IEC, International Organization for Standardization/International Electrotechnical Commission; SASC, Saud Abdulaziz Al Shalan Company

Site	Laboratory type/setting	Region	Approximate users	Baseline accreditation status	Previous document-control method
1	Toxicology	Jazan	30	CAP	Manual and previously used Medialab
2	Medical	Makkah	130	CAP and CBAHI accredited	Previously used MediaLab
3	Forensic	Aseer	60	CAP accredited	Manual and previously used cloud-based MediaLab
4	Medical	Riyadh	200	CAP and CBAHI accredited	Manual
5	Toxicology	Makkah	120	CAP	Previously used MediaLab and manual
6	Poison Control Centre	Riyadh	80	CAP	Manual
7	Medical and Toxicology	Saudi Arabia	30	CAP, CBAHI, and ISO 15189/ISO 17025 accredited	Manual

Implementation records confirmed active onboarding, configuration, and document-control use across these sites. By the study cut-off, six of the seven pioneer sites were fully active, and one remained in partial implementation status. Across the seven sites, the median number of active users was 80 (range: 30-200), the median number of configured departments was 10 (range: 5-15), and the median number of migrated or newly created controlled documents was 76 (range: 10-274) across the six sites with document data available. Site-level implementation metrics are presented in Table 4.

**Table 4:** Site-level implementation summary

Site	Active users	Departments configured	Documents migrated/created	Implementation status
Lab 1	30	10	124	Active
Lab 2	130	7	82	Active
Lab 3	60	15	274	Active
Lab 4	200	10	70	Active
Lab 5	120	10	50	Active
Lab 6	80	9	10	Active
Lab 7	30	5	-	Partial

Across pilot sites, the platform was used to configure organizational hierarchies, establish departmental document structures, onboard users, migrate or create controlled documents, and support internal audit and accreditation-readiness workflows. One early deployment site completed CAP accreditation activities during the period of Makeen DCS use, indicating that the platform could be used operationally in a regulated accreditation-oriented environment. However, the present study does not support causal attribution of that accreditation outcome to the platform.

Objective operational metrics such as document retrieval time, approval turnaround time, overdue review rates, and document-related nonconformities were not uniformly collected during this early implementation phase and are therefore not reported here as quantitative outcome measures. Accordingly, the early deployment findings should be interpreted as evidence of implementation feasibility, controlled document activation, and initial uptake rather than as proof of workflow-effectiveness impact.

User evaluation and feedback

The platform was further evaluated through an internally developed implementation-feedback survey completed by eight purposively selected respondents drawn from pilot sites. Respondents represented both leadership and operational roles, including laboratory directors, quality managers, document-control personnel, and staff users (Table 5).

**Table 5:** Respondent profile for user evaluation

Role	Count
Laboratory directors	2
Quality managers	2
Document control personnel	1
Staff users	3
Total	8

In this small implementation-linked sample, user feedback was favorable. The average satisfaction score across usability, accessibility, and compliance-related functionality was 9/10. Seven of eight respondents (87.5%) stated that they would recommend the Makeen DCS to other facilities. All eight respondents (100.0%) agreed or strongly agreed that the platform improved inspection readiness, and all eight (100.0%) agreed or strongly agreed that it reduced manual or paper-based workload. Positive feedback on training and support was reported by all respondents. These findings describe early acceptability within pilot implementation sites and should not be interpreted as independent or generalizable evidence of effectiveness (Table 6).

**Table 6:** User evaluation results

Survey item	Numerator/denominator	Value	Notes
Would recommend the Makeen DCS to other facilities	07/08	87.50%	Descriptive implementation-feedback item
Improved inspection readiness (agree or strongly agree)	07/08	100.00%	All respondents agreed or strongly agreed
Reduced manual/paper workload (agree or strongly agree)	07/08	100.00%	All respondents agreed or strongly agreed
Training feedback positive	07/08	100.00%	All respondents reported a positive training experience
Support feedback positive	07/08	100.00%	All respondents reported positive support experience
Average satisfaction score	Not count-based	09/10	Reported as the mean overall satisfaction

Respondents also reported that the platform was being used in support of multiple accreditation and audit frameworks, including CAP, CBAHI, ISO 15189/17025, and Ministry of Health audits. Because more than one framework could be selected by each respondent, these values are reported as multi-select mentions rather than denominator-based percentages.

Free-text comments emphasized the practical value of Arabic usability, centralized document visibility, and easier preparation for inspections. Because the survey sample was small and purposively selected from implementation sites, these findings should be interpreted as descriptive early adoption observations.

Respondents also indicated that the platform was being used in support of multiple accreditation and audit frameworks, including CAP (five mentions), CBAHI (two mentions), ISO 15189/17025 (two mentions), and Ministry of Health audits (two mentions). Because more than one framework could be selected by each respondent, these values represent multi-select mentions rather than denominator-based percentages. Free-text feedback from leadership and operational users emphasized Arabic usability, centralized document visibility, and easier preparation for inspections. Because the survey sample was small and purposively drawn from implementation sites, these findings should be interpreted as descriptive early adoption observations rather than generalizable effectiveness evidence.

## Discussion

This study provides early descriptive evidence on the development, standards alignment, internal functional validation, and pilot implementation of the Makeen DCS as a Saudi-built laboratory document control platform. The strongest findings are that the platform was developed against relevant accreditation and governance requirements, that 126 internally defined functional test cases were executed with all cases ultimately passing after correction and retesting, and that pilot deployment was achieved across seven purposively selected sites with six fully active by study cut-off. In addition, a small implementation-feedback sample reported a favorable early user experience. Taken together, these findings support internal functional readiness and early implementation feasibility. They do not, however, establish operational effectiveness, accreditation benefit, or broader system impact.

That distinction is central to interpreting the study appropriately. Document control is a core component of laboratory quality systems because it underpins standardization, traceability, accountability, and inspection readiness [1-3,14-17]. From that perspective, the functions demonstrated by the Makeen DCS, including structured approval workflows, version control, audit trails, searchable retrieval, and role-based access, are conceptually consistent with recognized principles of laboratory quality management and information governance [14-18]. However, conceptual alignment with quality-system principles should not be conflated with demonstrated downstream impact. The present study shows that the platform could be designed, validated internally, and deployed in real laboratory settings; it does not show that it improved laboratory performance in a measurable comparative sense.

The findings are most appropriately interpreted as feasibility observations. The standards-mapping exercise and internal validation results suggest that the system was functionally configured to support key document-control requirements drawn from CAP, ISO 17025, ISO 15189:2022, AABB, CBAHI, FDA Part 11, and PDPL-oriented governance considerations. The multisite deployment data further show that implementation was operationally possible across different Saudi laboratory contexts, including medical, toxicology, forensic, and poison control settings. Those are meaningful contributions, particularly in a context where local language needs, domestic hosting expectations, and accreditation-related workflows are important practical considerations [8-13,25-27]. Even so, feasibility should remain the principal claim.

The user-evaluation findings also require cautious interpretation. Although the survey responses were favorable, the sample was extremely small (n=8), purposively selected, and drawn from sites already linked to implementation. The survey was internally developed and descriptive, and the findings were perception-based rather than supported by independent operational metrics. Accordingly, the survey results are best understood as signals of early acceptability and implementation experience, not as reliable evidence of generalized user acceptance or effectiveness. This distinction directly matters because favorable satisfaction, recommendation, or perceived readiness data may coexist with implementation bias, selection bias, and developer-led evaluation effects.

The absence of objective comparative or longitudinal performance metrics further limits inference. The study did not include standardized pre-post data on document retrieval time, approval turnaround time, overdue review burden, document-related nonconformities, audit deficiencies, or accreditation outcomes. As a result, any interpretation that extends to improved efficiency, improved compliance performance, or reduced quality events would exceed the available evidence. One site completed CAP accreditation activities during the implementation period, but that observation is descriptive only and cannot be attributed causally to the platform. In this respect, the current manuscript is stronger when it explicitly distinguishes between implementation feasibility and measurable organizational impact.

Within those boundaries, the study still has contextual value. Saudi laboratory and accreditation literature has highlighted the importance of standardization, structured documentation, staff readiness, IT support, and governance capacity in achieving quality-system goals [8-11]. The present study contributes to that literature by describing an early implementation experience of a locally developed document-control platform configured around Saudi laboratory conditions, including bilingual Arabic-English operation and Saudi-hosted deployment. This does not demonstrate superiority over other systems, but it does indicate that a locally developed platform can be configured to reflect local accreditation and governance priorities while remaining technically usable across more than one site.

The study also has several strengths. It used a requirements-first, standards-based development approach rather than presenting the software only as a technical product. It explicitly mapped system functions to relevant accreditation and governance domains. It moved beyond prototype description by reporting internal validation, deployment status across multiple sites, and site-level implementation metrics. It also addressed a context not well represented in the manuscript's cited literature: the early implementation of a Saudi-built laboratory document control platform that combines accreditation-oriented workflow logic, bilingual usability, and domestic hosting.

The limitations, however, are substantial. Validation was internal and developer-led, with no independent external assessor. The implementation survey sample was very small, purposively selected, and linked to sites already participating in deployment. No comparator group was included. Objective operational performance indicators were not uniformly collected, so claims regarding efficiency or compliance improvement cannot be made. In addition, the authors' dual role as platform developers and evaluators introduces the possibility of bias and should be considered when interpreting both the validation and acceptability findings. Generalizability is also limited because the study was conducted in Saudi laboratory settings with specific accreditation, language, and data-governance conditions.

Future work should therefore move from feasibility to independent effectiveness evaluation. Priority areas include external validation, multicenter longitudinal assessment, and standardized measurement of implementation and performance indicators, such as document retrieval time, approval turnaround time, overdue review burden, inspection-preparation effort, document-related nonconformities, and accreditation-related deficiencies. It would also be valuable to assess interoperability with broader laboratory information ecosystems and to evaluate whether the platform's implementation is associated with measurable operational benefit over time [4-6,21,23].

In summary, the present findings support a restrained conclusion: The Makeen DCS was internally validated and feasibly implemented across multiple pilot laboratory settings, and a small implementation-linked sample reported a favorable early experience. These observations justify continued study of the platform, but they do not yet justify claims of demonstrated effectiveness, broad generalizability, or system-wide impact.

## Conclusions

The Makeen DCS is a Saudi-developed laboratory document control platform designed to support accreditation-oriented document governance through standards-based workflow design, bilingual Arabic-English functionality, role-based control, audit trails, and Saudi-hosted deployment options. In this study, the platform showed internal functional readiness, pilot deployment across multiple sites, and favorable early user feedback within a small purposive sample. These findings support feasibility and early acceptability only. They do not establish effects on operational efficiency, document-related nonconformities, accreditation outcomes, or patient-level outcomes. Broader claims regarding effectiveness or wider applicability should await independent, multicenter, and longitudinal evaluation.
